# Misleading pustular plaques of the lower limbs during Crohn's disease: two case reports

**DOI:** 10.1186/1752-1947-1-109

**Published:** 2007-10-03

**Authors:** David Farhi, Paul Duriez, Selim Aractingi, Jacques Cosnes, Kiarash Khosrotehrani

**Affiliations:** 1 AP-HP, Hôpital Tenon, Department of Dermatology, Paris F-75020, France; 2 AP-HP, Hôpital Saint Antoine, Department of Pathology, Paris F-75012, France; 3 AP-HP, Hôpital Saint Antoine, Department of Gastroenterology, Paris F-75012, France

## Abstract

**Background:**

Extraintestinal manifestations of Crohn's disease may involve the skin, the eyes, the genital mucosa, and the joints. Dermatoses associated with Crohn's disease include neutrophilic dermatoses, erythema nodosum, granulomatous dermatitis, blistering dermatoses, and non-specific skin manifestations. Cutaneous Crohn's disease is characterized by skin non-caseating epithelioid granulomatas with giant cells, remote from the gastrointestinal tract. We report herein two new cases.

**Observations:**

On both patients, differential diagnosis of neutrophilic dermatoses and infectious disease were evoked, and antimicrobial agents were introduced in one of them. Given the atypical presentation, the final diagnosis of cutaneous Crohn's disease could only be made with histological examination. In patient 1, the plaques decreased in size and infiltration by more than 75% after 3 weeks of treatment with bethametasone dipropionate 0.05% cream. In patient 2, the plaques decreased by more than 50% after 6 weeks of treatment with prednisolone (45 mg/day) and azathioprine (100 mg/day).

**Discussion:**

Cutaneous Crohn's disease may present as dusky, erythematous, infiltrated, and ulcerated plaques and nodules. Female-to-male sex ratio is about 2, and the mean age at onset is 35. Recurrently, the hypothesis of a skin mycobacterial or fungal infection greatly delays proper treatment. Rarity of cutaneous Crohn's disease hampers therapeutic assessment in controlled trials. Thus, available literature is limited to case reports and sparse small series, with contradictory results. These reports are subject to publication bias, and no definite evidence-based recommendations can be made on the most adequate therapeutic strategy.

## Background

Crohn's disease is characterized by a chronic relapsing transmural granulomatous inflammation that may involve any part of the digestive tract from mouth to anus, though mostly found in the ileum, the cecum, and the colon. It involves a type-1 helper T lymphocytes – mediated immune reaction of unknown etiology, which may result from an interaction between environmental factors such as enteric bacteria, and a genetic susceptibility [1,2].

Extraintestinal manifestations of Crohn's disease may involve the eyes, the skin, the genital mucosa, and the joints. Associated distant skin manifestations are polymorphous, and occur in 14 to 44% of patients with Crohn's disease [3,4]. They can be classified in two categories depending on whether they are a direct ("metastatic") manifestation of the granulomatous disease or simply associated with it. The range of skin manifestations associated with Crohn's disease includes neutrophilic dermatoses such as pyoderma gangrenosum and Sweet's syndrome, various panniculitis such as erythema nodosum, granulomatous dermatitis exemplified by palisading granulomatous dermatitis and necrobiosis lipoidica [5], blistering dermatoses such as epidermolysis bullosa acquisita and erythema multiform, and non-specific skin manifestations such as adverse drug reactions and malabsorption-associated dermatologic changes.

Cutaneous Crohn's disease, also called metastatic Crohn's disease, is a rare manifestation characterized by a prominent non-caseating granulomatous inflammation developing in the skin. This manifestation is by definition remotely located from the gastrointestinal tract with non specific clinical features, and often misleads clinicians and delays adequate therapy [6]. We report herein two cases of cutaneous Crohn's disease located on the legs.

### Patient 1

**Figure 1:**
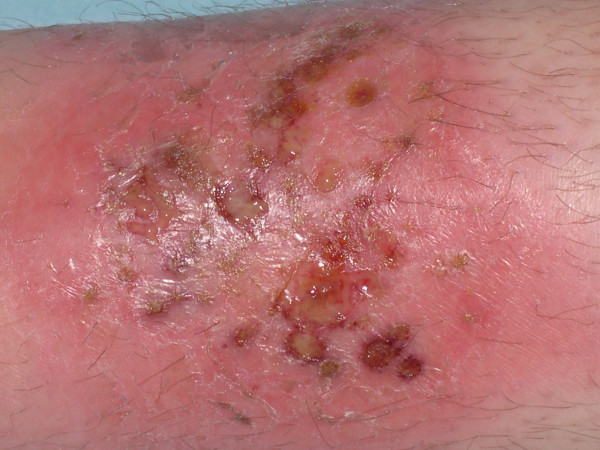
Anterior aspect of the right leg of patient 1: superficial erythematous ulcerated plaque, covered by pustules.

**Figure 2:**
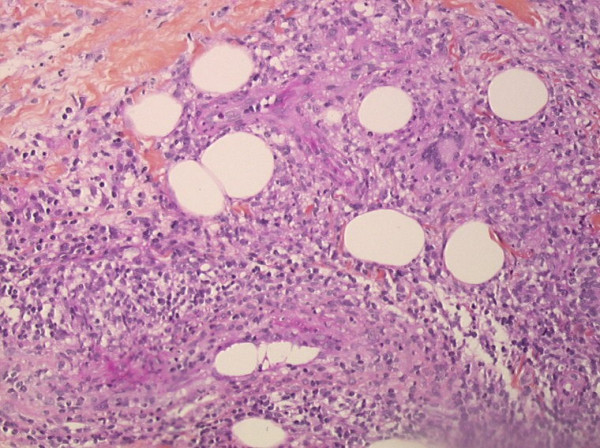
Histological examination of a skin biopsy specimen of the plaque showed on figure 1: lymphohistiocytic hypodermal infiltrate, associated with non-caseating multinucleated giant cells granulomas (Hematoxylin-eosin Strain; original magnification ×100).

A 25-year-old woman was referred for cutaneous lesions on the right leg, developing a month after she returned from Mali. She had been diagnosed with Crohn's disease at age 22, and was maintained in complete remission under mesalazine. On physical examination, she had pustular plaques of the right leg (figure 1), with two satellite lesions of smaller size. There was no sign of digestive relapse. She had already been treated with oxacilline (3 g/day, 7 days), without any improvement. Clinical hypotheses were Sweet's syndrome, pyoderma gangrenosum, pustular psoriasis, bacterial or parasitic skin infection, and mycetoma. Repeated bacteriological and mycological investigations were negative. Skin histology showed a lymphohistiocytic hypodermal infiltrate, associated with non-caseating multinucleated giant cells granulomas (figure 2). Antimicrobial agents were discontinued, and bethametasone dipropionate 0.05% cream was initiated. The plaques decreased in size and infiltration by more than 75% within 3 weeks.

### Patient 2

A 51-year-old woman presented with papules of the calves, evolving since 3 weeks. She had Crohn's disease since the age of 21. A flare of Crohn's ileocolitis was developing for five months, with diarrhea and abdominal pain. On clinical examination, she had asymptomatic, infiltrated, erythematous and ulcerated, papules and plaques of the calves, and a right keratoconjonctivitis. After clinical examination, diagnostic hypotheses were Sweet's syndrome, erythema nodosum, pyoderma gangrenosum, and mycobacterial, fungal or bacterial skin infection. Repeated bacteriological and mycological investigations were negative. Histological examination showed a necrotizing vasculitis characterized by thromboses and necroses of dermal blood vessels associated with a dermal infiltrate of pycnotic neutrophilic granulocytes. The hypodermis enclosed non-caseating epithelioid granulomata with multinucleated giant cells consistent with a cutaneous Crohn's disease. Ziehl, Grocott and PAS staining failed to identify infectious agents. Considering the ongoing flare of Crohn's ileocolitis, a systemic treatment was introduced. The plaques decreased by more than 50% after 6 weeks of treatment with prednisolone (45 mg/day) and azathioprine (100 mg/day).

## Discussion

We here report two cases of cutaneous Crohn's disease, where the diagnosis proved difficult due to the lack of specificity of the cutaneous clinical signs. In both cases, the definite diagnosis was obtained – after ruling out an infectious disease – by histology, which shows a granulomatous infiltrate.

Cutaneous Crohn's disease was first described in 1965, and was subsequently named "Metastatic Crohn's disease" in 1970 [7]. The ambiguous term "metastatic" – which may unfortunately generate confusion with the notion of "malignancy" – refers to the fact that skin lesions are remote from the gastrointestinal tract, from which they are separated by normal skin. Considering the inflammatory nature of the disease, we – as others [7] – emphasize that the term "metastatic" should be abandoned and replaced by "cutaneous".

Cutaneous Crohn's disease typically presents clinically as dusky, erythematous, infiltrated, and sometimes ulcerated plaques and nodules. Approximately two thirds of the patients are female, and the mean age at onset is 34.5 (range: 5 – 71) [6]. Interestingly, there does not seem to be any correlation between the intestinal and the cutaneous activity of Crohn's disease [6,7] as it was the case for patient 1. These skin lesions are predominantly found in the genital region and on lower extremities but have as well been described on the face, the abdomen, the perineum, the flexural areas (submammary folds, retroauricular region, groin, and abdominal folds) [6,8].

Given the variable topography and presentation, clinical differential diagnoses are plentiful. Especially when gastrointestinal Crohn's disease has not been previously identified, clinical diagnosis can be quite subtle. Conversely, even when gastrointestinal Crohn's disease has been previously diagnosed, cutaneous Crohn's disease can be clinically confused with various differential diagnoses, such as pyoderma gangrenosum or mycobacterial skin infections, that were both considered in our patients. However, in all clinical contexts as in our observations, establishing definite diagnosis requires the histological examination of a skin lesion, which typically shows dermal and/or hypodermal non-caseating epithelioid granulomata, with scattered multinucleated giant cells, and a crown of peripheral lymphocytes and plasma cells. A perivascular lymphomononuclear infiltrate may be associated. Frequently, the consideration of a skin mycobacterial or fungal infection greatly delays proper treatment; it is of note that these infectious diseases may as well occur during the course of Crohn's disease, either coincidentally or as an effect of immunosuppressive therapies. Ruling out infections, usually requires bacteriological and mycological workup. In this context, the use of polymerase chain reaction on a skin biopsy may help shorten the time to exclude some infectious agents, including mycobacteria.

The treatment of cutaneous Crohn's disease remains unsatisfactory. Furthermore, its scarcity hampers therapeutic assessment in controlled trials. Thus, available literature is limited to case reports and sparse small series. Successful treatments have been reported with métronidazole [8]. Contradictory results have been reported with mesalazine [9], mycophenolate mofetil [10], steroids [8,9], azathioprine [9], sulphasalazine [8], and infliximab [9]. Surgical excision of the affected intestine seems not to improve cutaneous Crohn's lesions. However, all these reports are subject to publication bias, and no definitive evidence-based statement can reasonably be made on the most adequate management strategy for cutaneous Crohn's disease. In our cases, corticosteroids administered either topically or systemically were greatly efficient.

## Conclusion

We here describe two cases of cutaneous Crohn disease of the lower legs. A better knowledge of the possibility of distant Crohn manifestations should improve the delay of adequate management of cutaneous complications of this debilitating disease.

## Competing interests

The author(s) declare that they have no competing interests.

## Authors' contributions

All authors contributed to each stage of this work.

This means that DF, PD, SA, JC and KK all have: (1) made substantial contributions to conception and design, or acquisition of data, or analysis and interpretation of data; (2) been involved in drafting the manuscript or revising it critically for important intellectual content; and (3) given final approval of the version to be published.
